# Predicting bird song from space

**DOI:** 10.1111/eva.12072

**Published:** 2013-05-08

**Authors:** Thomas B Smith, Ryan J Harrigan, Alexander N G Kirschel, Wolfgang Buermann, Sassan Saatchi, Daniel T Blumstein, Selvino R de Kort, Hans Slabbekoorn

**Affiliations:** 1 Department of Ecology and Evolutionary Biology, University of California Los AngelesLos Angeles, CA, USA; 2 Center for Tropical Research, Institute of the Environment and Sustainability, University of California Los AngelesLos Angeles, CA, USA; 3 Department of Biological Sciences, University of Cyprus NicosiaCyprus; 4 Department of Zoology, Edward Grey Institute, University of OxfordOxford, UK; 5 Jet Propulsion Laboratory, California Institute of Technology PasadenaCA, USA; 6 Division of Biology and Conservation Ecology, School of Science and the Environment, Manchester Metropolitan University ManchesterUK; 7 Behavioural Biology, Institute of Biology, Leiden UniversityLeiden, The Netherlands

**Keywords:** anthropogenic effects, avian song, behavioral ecology, random forests, remote sensing, reproductive isolation, spatial heterogeneity

## Abstract

Environmentally imposed selection pressures are well known to shape animal signals. Changes in these signals can result in recognition mismatches between individuals living in different habitats, leading to reproductive divergence and speciation. For example, numerous studies have shown that differences in avian song may be a potent prezygotic isolating mechanism. Typically, however, detailed studies of environmental pressures on variation in animal behavior have been conducted only at small spatial scales. Here, we use remote-sensing data to predict animal behavior, in this case, bird song, across vast spatial scales. We use remotely sensed data to predict the song characteristics of the little greenbul (*Andropadus virens*), a widely distributed African passerine, found across secondary and mature rainforest habitats and the rainforest-savanna ecotone. Satellite data that captured ecosystem structure and function explained up to 66% of the variation in song characteristics. Song differences observed across habitats, including those between human-altered and mature rainforest, have the potential to lead to reproductive divergence, and highlight the impacts that both natural and anthropogenic change may have on natural populations. Our approach offers a novel means to examine the ecological correlates of animal behavior across large geographic areas with potential applications to both evolutionary and conservation biology.

## Introduction

Acoustic characteristics of bird song, such as the temporal and spectral structure, may vary among habitats as a result of environmental pressures imposed by natural selection (Morton 1975; Wiley and Richards 1982; Slabbekoorn and Smith 2002a). Numerous studies have shown that differences in avian song may be a potent prezygotic isolating mechanism that can drive divergence and speciation (Price 2008; Uy et al. 2008; Podos 2010). Song variation may be driven by differences in forest structure (Wiley and Richards 1982), acoustic competition with other birds (Kirschel et al. 2009a), or background noise (Slabbekoorn and Smith 2002a; Price 2008). Recent research has also demonstrated that human-altered environments can lead to changes in avian song (Slabbekoorn and Peet 2003; Slabbekoorn and den Boer-Visser 2006; Halfwerk and Slabbekoorn 2009; Kirschel et al. 2009a,b). For example, low-frequency traffic noise in urban areas has been shown to cause birds to increase their minimum song frequencies (Nemeth et al. 2013). Such anthropogenic related song divergence may lead to signal mismatches between populations living in natural and human-altered environments, thereby altering interactions and territory acquisition among males, and eventually causing disruption of gene flow between the habitats. (Mockford and Marshall 2009; Ripmeester et al. 2010).

Distinguishing between habitats has now been made possible using spaceborne sensors that capture ecosystem characteristics that assist in explaining their biological function, health, and diversity (Calder 1973; Turner et al. 2003; Chambers et al. 2007; Saatchi et al. 2008). However, remote-sensing technology has never by itself been used to quantify and predict species' acoustic signaling across landscapes. To examine the utility of using remotely sensed data to predict animal behavior, we constructed models based on satellite observations of ecosystem structure and function and measurements of the song of little greenbuls (*Andropadus virens*), a common African rainforest passerine. We recorded vocalizations during field surveys across habitats, including those modified by humans. Because little greenbuls are found across much of equatorial Africa in a diversity of forest habitats, they provide an excellent test case for integrating quantitative data on acoustic behavior with detailed remote-sensing data on habitat characteristics.

Little greenbuls have a repertoire of four songtypes. Songtype I and II are characterized by simple chatter with limited frequency ranges, whereas songtypes III and IV are more complex. In a previous analysis, the two complex songtypes (III and IV) were found to differ significantly between rainforest and ecotone habitats in Cameroon and were used as indicators of differences in habitat structure and/or cultural similarities (Slabbekoorn and Smith 2002b). Sound-transmission properties of song showed no differences between habitats for the little greenbul-specific forest layers, but ambient noise differed greatly (Slabbekoorn 2004), and was considered a likely factor driving differences in song characteristics between populations (Slabbekoorn and Smith 2002b). In addition to song, morphological traits important to fitness also vary across the rainforest and rainforest-savanna ecotone (Smith et al. 1997, 2005). Song divergence across ecological gradients may lead to reproductive divergence and ultimately to speciation, if populations spaced along a gradient do not recognize divergent songs (Irwin et al. 2001; Slabbekoorn and Smith 2002a). Recent experiments demonstrate a significant reduction in the response of adult male greenbuls from the rainforest to playbacks of male ecotone–forest songs compared to other rainforest populations (Kirschel et al. 2011). This suggests that habitat-dependent song variation is perceptually important to little greenbuls and could play an important role in reproductive divergence (Slabbekoorn and Smith 2002b; Price 2008; Uy et al. 2008; Kirschel et al. 2009b; Podos 2010). Based on previous work, there are several predictions that can be made concerning greenbul song variation in relation to habitat differences. First, we predict that remote-sensing layers characterizing habitat variation will accurately explain variation observed in greenbul song characteristics. Second, greenbuls from mature forest and ecotone should differ greatly in song characteristics, as these environments represent the two extremes of the ecological range of this species. Third, greenbuls in anthropogenically altered sites should exhibit song characteristics intermediate between those of mature and ecotone habitats.

The objectives of this study were to: (i) evaluate the extent to which remote-sensing data alone can predict song characteristics of the little greenbul, (ii) determine whether distinct habitat types, such as primary rainforest, human-altered secondary forest, and ecotone, may harbor greenbul populations with unique song characteristics, and (iii) discuss some of the practical advantages and disadvantages of using remotely sensed data to examine the ecological correlates of avian song and predict evolutionary processes across large spatial scales.

## Materials and methods

### Song collection

**Figure 1:**
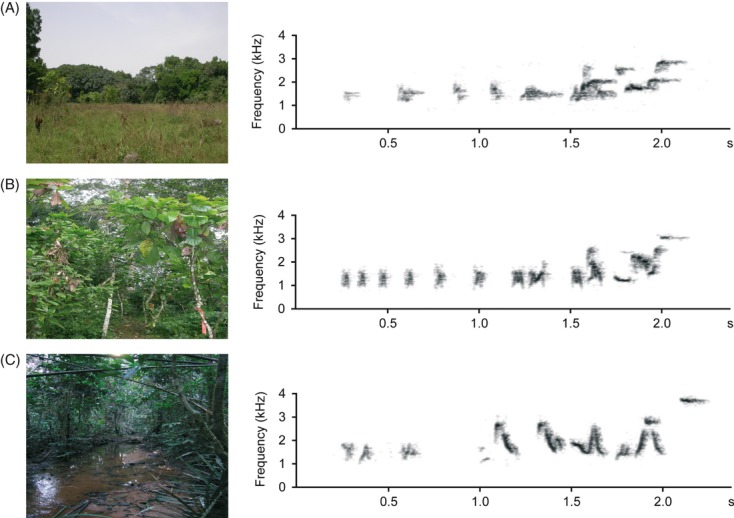
Examples of the three habitats and associated spectrograms of little greenbul songtype III. (A) Ecotone–forest habitat as seen from the savanna edge (Ngoundaba). (B) Degraded secondary rainforest (Nkwouak). (C) Mature rainforest (Zoebefam). Note the higher maximum frequency of the terminal note in the mature forest.

Little greenbuls were recorded singing along forest roads and trails at 24 sites in Cameroon between June 1998 and August 2009. We recorded a total of 2084 songs from 117 individual greenbuls at 24 sites (91% of the data comprised of 4–5 recordings from each of five individuals) in Cameroon (covering an approximately 336 762 km^2^ area) including undisturbed mature rainforest, anthropogenically degraded rainforests (or secondary forest), and ecotone forest (Fig. 1, Tables S1 and S2). This typically included five recordings of each songtype per individual. Recordings were collected using a Sennheiser (Wedemark, Germany) ME67 microphone either with a Sony (Tokyo, Japan) TCM-5000EV tape recorder with TDK (Garden City, New York, USA) SA90 tapes, or with a Marantz (Osnabrück, Germany) PMD 670 solid-state digital recorder recording at a 44.1 kHz sampling rate. We collected song recordings of singing males by walking along a trail or road and identifying each individual. In this way, it was easy to distinguish individual territories and to differentiate one singing male from another. Extra care that was taken to insure a given male was not recorded twice.

### Song analyses

Although each of the four songtypes were measured for acoustic variation, we focused our primary analyses on songtypes III and IV, the two most complex of the songtypes, because they were previously identified to be important in intraspecific communication between males and females, showed strong associations with environmental variables and were previously found to vary across sites (Slabbekoorn and Smith 2002b). Furthermore, although any part of the song may play a dual role in sex-specific communication, the longest and most elaborate songtype IV has been put forward as most likely to be important for intraspecific communication between males and females (Slabbekoorn and Smith 2002b), while playback experiments suggested that especially the shorter but conspicuous songtype III may be responsible for differences in responsiveness between males from different habitats (Kirschel et al. 2011).

Individual songs with a sufficiently good signal-to-noise ratio were clipped from the larger field recordings using Syrinx software (http://www.syrinxpc.com, John Burt). Each song file was then visually inspected for background noise showing any spectral and temporal overlap with little greenbul song. If any overlap was found, the file was excluded from further analysis. Background noise that did not show overlap with little greenbul song was filtered using Avisoft-SASLAB Pro software (Avisoft Bioacoustics, Berlin, Germany) to ensure that the little greenbul song was the sound with the highest amplitude in the song file. This was a prerequisite for using the Automatic Parameter Measurement (APM) command in Avisoft-SASlab. APM was then applied to detect little greenbul song notes (the smallest continuous sound in a song) using an amplitude threshold of −18 dB SPL relative to the maximum SPL in the sound file. This procedure ensured that measurements were standardized across all sound files. Within each detected song note, the following parameters were measured: start time, end time, entropy – measured as the mean of all spectra, maximum frequency – measured as the highest frequency exceeding threshold, minimum frequency – measured as the lowest frequency exceeding threshold, and peak frequency – measured at the sampling point with the highest amplitude. Song rate was calculated by dividing the song duration (calculated from the start and end times) by the number of notes in the song. We strove to include five individuals per site and up to five of each of the songtypes per individual. Statistical analyses were conducted on the mean value per songtype and per individual. Spectrogram settings for all analyses were: Fast Fourier Transform size = 512, Hamming window, bandwidth = 41 Hz, resolution = 31 Hz, temporal overlap = 87.5%. All measurements were then entered into Microsoft Excel (2007) from which we derived the following variables: song duration, song rate, average entropy of all song elements, and minimum, maximum and peak frequency of the song.

### Habitat characteristics

Habitat characteristics for each of the sites were inferred using optical passive and microwave active satellite that capture various aspects of habitat structure including canopy greenness, structure and moisture, and tree cover. Habitat characteristics were inferred using three spaceborne sensors: high resolution (∼250 m) MODIS (Moderate Resolution Imaging Spectroradiometer), high resolution (∼100 m) ALOS PALSAR (Advanced Land Observing Satellite Synthetic Aperture Radar), and coarse resolution (∼2.25 km) QuikSCAT scatterometer (QSCAT), and derived products (National Aeronautics and Space Administration, Washington, DC, USA) (Table 1).

**Table 1:** Satellite remotely sensed variables and derived products used, with brief description

Variable	Description
Microwave active	
QSCATM	Annual mean, measure of surface moisture/roughness, biomass
QSCATS	Annual stdev, measure of temporal variations in surface moisture/roughness, biomass
SRTMM	Elevation, mean
SRTMS	Elevation, standard deviation, measure of ruggedness
ALOS HH	HH polarization, measure of surface moisture/roughness, biomass
ALOS HV	HV polarization, measure of surface moisture/roughness, biomass
Optical passive	
MODIS B1	620–670 nm band range, photosynthetic activity (chlorophyll *b*)
MODIS B2	841–876 nm band range, internal leaf structures
MODIS B3	459–479 nm band range, photosynthetic activity (chlorophyll *a*)
MODIS B7	2105–2155 nm band range, leaf water content
Derived products	
ALOS RFDI	Rain Forest Degradation Index (ALOS HH/HV), biomass
TREE	MODIS-derived vegetation continuous field (VCF)
NDVI	Normalized Difference in Vegetation Index, (MODIS B2-B1/B1 + B2), photosynthetic activity
NDII	Normalized Difference in Infrared Index, (MODIS B2-B7/B2 + B7), leaf water content (decreasing values correspond to higher leaf water content)

The original satellite data have various native spatial (250 m–2.25 km) and temporal resolutions (4d-month), and we aggregated (pixel aggregate)/downscaled (nearest neighbor) all data to a common 1 km spatial grid on which tree regressions were applied. For consistency, all satellite data correspond to measurements from the year 2001, with the exception of ALOS layers, which are based on measurements from 2007.

Continuous remote-sensing data collected and evaluation of sites on the ground were used to classify habitats into three major types: primary forest, secondary forest, and ecotone habitats (Fig. 1). Sites were classified into one of these three habitat types by researchers in the field, and these classifications were later confirmed using remote-sensing data. These habitat classifications have been used previously to broadly define the extreme differences in habitat seen within the little greenbul habitat range (Slabbekoorn and Smith 2002b; Smith et al. 2008). These habitat classifications were not used in regression tree and random forest models (see below). However, they were used in pairwise comparisons of song characteristics across major habitat types to determine whether anthropogenic activity in forested sites may be associated with differences in songtype characteristics.

### Regression trees and random forests

To determine which environmental parameters (predictors) best explained observed variation in acoustic features (responses), we used regression trees in ‘tree’ (Liaw and Wiener 2002) and iterations of these in Random Forest (Breiman 2001) using the R statistical package (R Foundation for Statistical Computing 2011). Regression tree models partition the variables via a binary recursive approach to measure the relative importance of predictor variables in explaining the variation in the response variable. Random forest methods incorporate a large number of randomly constructed regression trees (both the suite of predictors and the records are randomly selected). New records (those not included in the training model) are then predicted using the average prediction across all trees, the accuracy of which are then used to evaluate model performance. Neither of these partitioning methods requires the use of any particular model (which might be difficult to assign given a behavioral variable such as song frequency), nor do they require normalized data, and have consistently outperformed traditional regression procedures on a number of data sets (Breiman 2001). Using these models, and continuous layers of environmental predictors, it is possible to spatially predict response variable characteristics even in areas that have not been sampled.

Both accuracy and precision of predictions were verified internally within the model (by using a bootstrapped bagging approach embedded in the random forest algorithm) and externally (by using independent test sites). The internal verification used subsets of records iteratively to construct model, test the accuracy, and improve the predictive model (Breiman 2001). Quantitative results reported from the optimal model reflect this internal cross-validation approach (Figures S5–S8). After optimizing the predictive model using song data collected between 1998 and 2007, we then analyzed songs recorded from three new independent sites subsequently sampled in 2009 (Table S2) to further validate the results. The standard error of estimates associated with predictive models for various songtypes and frequencies were low and showed no directionality or bias, particularly across all frequencies for songtype III maximum frequency and songtype IV minimum frequency, the two song characteristics whose variation was explained most by remote-sensing data reflecting habitat differences.

Regression trees and random forests were applied in the following manner: regression trees were performed on each song characteristic for each songtype (Figures S1–S4), and with every remotely sensed and derived variable listed in Table 1 included as input. Because certain records may bias model predictions, or predictors may be correlated (as is the case when comparing raw remote-sensing layers and those that are derived from the same layers), we ran 5000 regression trees, randomly selecting both input predictors and response records to construct a random forest (Breiman 2001). We estimated the importance of each variable by comparing the increase in the mean standard error when that variable was removed from models [Figures S5–S8, (Breiman 2001)]. In order to maximize the percent of response variable explained while minimizing the number of correlated variables, we selected the three or four (depending on number of correlated variables appearing at the top of the list) most important variables to use as input for the construction of the final model. Models of these songtype characteristics were verified according to two criteria: using a subset of initial samples left out for testing purposes (as part of the random forest algorithm), and in subsequent collection of data at three novel locations at later dates 2009; once we performed this evaluation, data collected from these three sites were included as response records in final model construction.

## Results

### Differences in song characteristics across varying ecological conditions

Results revealed that combinations of remotely sensed variables describing habitat characteristics explained large percentages of the variation in songtypes III and IV of the little greenbul. For example, remote-sensing variables explained 66% of the variation in maximum frequency of songtype III and 45% of the variation in minimum frequency of songtype IV.

**Figure 2:**
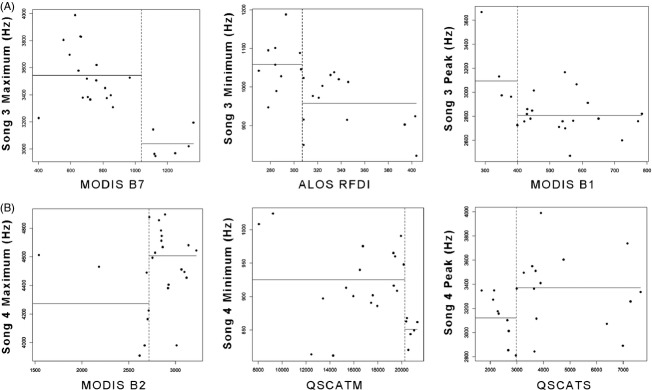
Environmental predictors of variation in songtype III and IV characteristics. Dotted line indicates the value of the environmental variable where regression tree has bifurcated the data to minimize the within-group variation. Horizontal solid lines indicate the mean song frequency within each of these groups. Shown are the relationships between the top environmental predictor and indicated songtype characteristic: (A) Songtype III characteristics were explained by measures of surface reflectance, with higher values (indicating lower biomass) associated with higher song III maximum frequencies and peak amplitudes, and by extent of deforestation, with lower minimum frequencies in more degraded areas. (B) Songtype IV characteristics were best explained by reflectance values (Moderate Resolution Imaging Spectroradiometer B2), and measures of surface moisture (QSCAT).

For songtype III, the best predictor of variation in maximum frequency was a remote-sensing layer capturing reflectance characteristics (MODIS B7, a measure of leaf water content; Table 1), capturing 56% of the total variation in max frequency, with individuals in more forested areas (lower reflectance) singing at higher maximum frequencies (Fig. 2A). A recently developed layer capturing levels of deforestation, the ALOS Rainforest Degradation Index (RFDI), best explained variation in songtype III minimum frequency, capturing 24% of total variation, with lower minimum frequencies found at sites with more forest degradation (Fig. 2A). The use of both higher maximum and higher minimum frequencies in songtype III in forested sites suggests that greenbuls are singing songs that are elevated in frequency across their entire frequency range as compared to less forested regions. This observation was also supported by the fact that the highest songtype III peak amplitude was recorded in forested regions (Fig. 2A). Overall, a large proportion of the variation in physical characteristics of songtype III was explained by remote-sensing layers (Mean percent variation explained for six song characteristics of songtype III = 38.5%, Figure S7).

Remote-sensing layers also explained several song characteristics of songtype IV, including maximum and minimum frequency, song rate, and entropy of this songtype. A measure of reflectance (MODIS B2) was found to have a positive association with songtype IV maximum frequency, with higher max frequencies recorded in regions with higher reflectance values (and therefore lower biomass, Fig. 2B). This variable, along with measures of elevation (Figure S8), captured 26% of the total variation in songtype IV maximum frequency. For songtype IV, minimum frequency captured 41% of the total variation with lower minimum frequencies recorded in areas with higher reflectance (as measured by QSCATM, and corresponding to areas with lower biomass, Fig. 2B). In contrast, environmental variables explained only 7% of the variation in peak frequency in songtype IV (Figs 2B, S8) suggesting that for at least some features, broad ecological differences between habitats may not be best at explaining variation in songtype characteristics. Despite this lack of explanatory power for some characteristics, a fair amount of the variation in physical characteristics of songtype IV was captured by the remote-sensing variables used (mean percent variation explained for six song characteristics of songtype IV = 25.5%, Figure S8).

Many song characteristics, including those from songtypes I and II, had large differences in spectral and temporal characteristics among the habitat types (Figures S1–S8). Consistent patterns among songtypes suggest that higher maximum frequencies are common among more forested areas, and more open areas with higher levels of deforestation, and higher reflectance values are regions with lower maximum (and lower minimum) frequencies (Figures S1–S8).

### Song variation across the landscape

**Figure 3:**
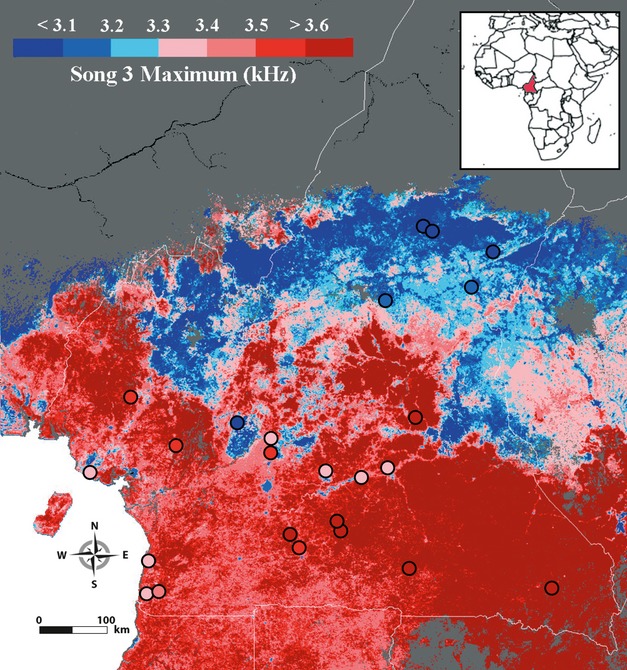
Predictive map for songtype III maximum frequency. Environmental variables explained 66% of the variation in songtype III maximum frequency. Sampling was based on 24 sample sites where song was recorded. Circles represent recording locations, and colors of those circles represent average observed songtype III maximum frequency at that location.

**Figure 4:**
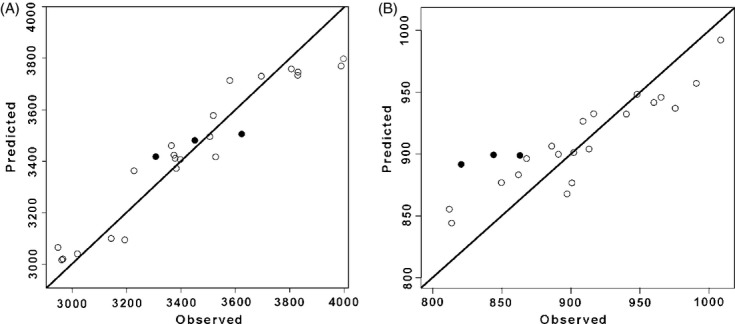
Accuracy of observed versus predicted values of (A) songtype III maximum and (B) songtype IV minimum frequency (in Hertz) in little greenbuls in Cameroon. Line represents perfect predictions. Open circles represent sampled sites that were included in model construction (Standard Error of the Estimate = 11.9 and 26.5 Hz, respectively), closed circles represent sites that were visited in August 2009 and used to ground-truth model statements (Standard Error of the Estimate = 19.4 and 46.3 Hz, respectively).

Given the success of remote-sensing layers in explaining song characteristics of the little greenbul, and the extraordinary amount of variation explained by songtype III maximum frequency, the relationship between habitat and songtype III maximum frequency was applied to unsampled portions of the little greenbul range to spatially predict this song variable across southern Cameroon and parts of surrounding countries. Typically, predictions from spatial models tend to perform well near sampled regions, and this is true of our data (St. Estimate of Error = 11.9 Hz). However, a test of our model at new sites not used in model construction also resulted in accurate predictions (St. Estimate of Error = 19.4 Hz). Spatial predictions of songtype III maximum frequency suggest major breaks between habitat types within Cameroon, and suggest that this song characteristic is tightly linked to ecological conditions (Fig. 3). Songtype III maximum frequency is also the character identified as the most likely to explain variation in responses to playback experiments (Kirschel et al. 2011) and is largely explained by satellite variables that measure the degree of canopy openness and forest canopy biomass. Regions with songs of higher maximum frequencies were characterized by closed canopy and higher canopy biomass (Fig. 3). A similar spatial prediction of songtype 4 minimum frequency (not shown) also tracked differences in surface reflectance, although error estimates were higher for this model (St. Error of Estimate = 26.5 Hz for trained sites, and 46.3 Hz for new sites not used in models, Fig. 4).

### Song in human-altered environments

**Figure 5:**
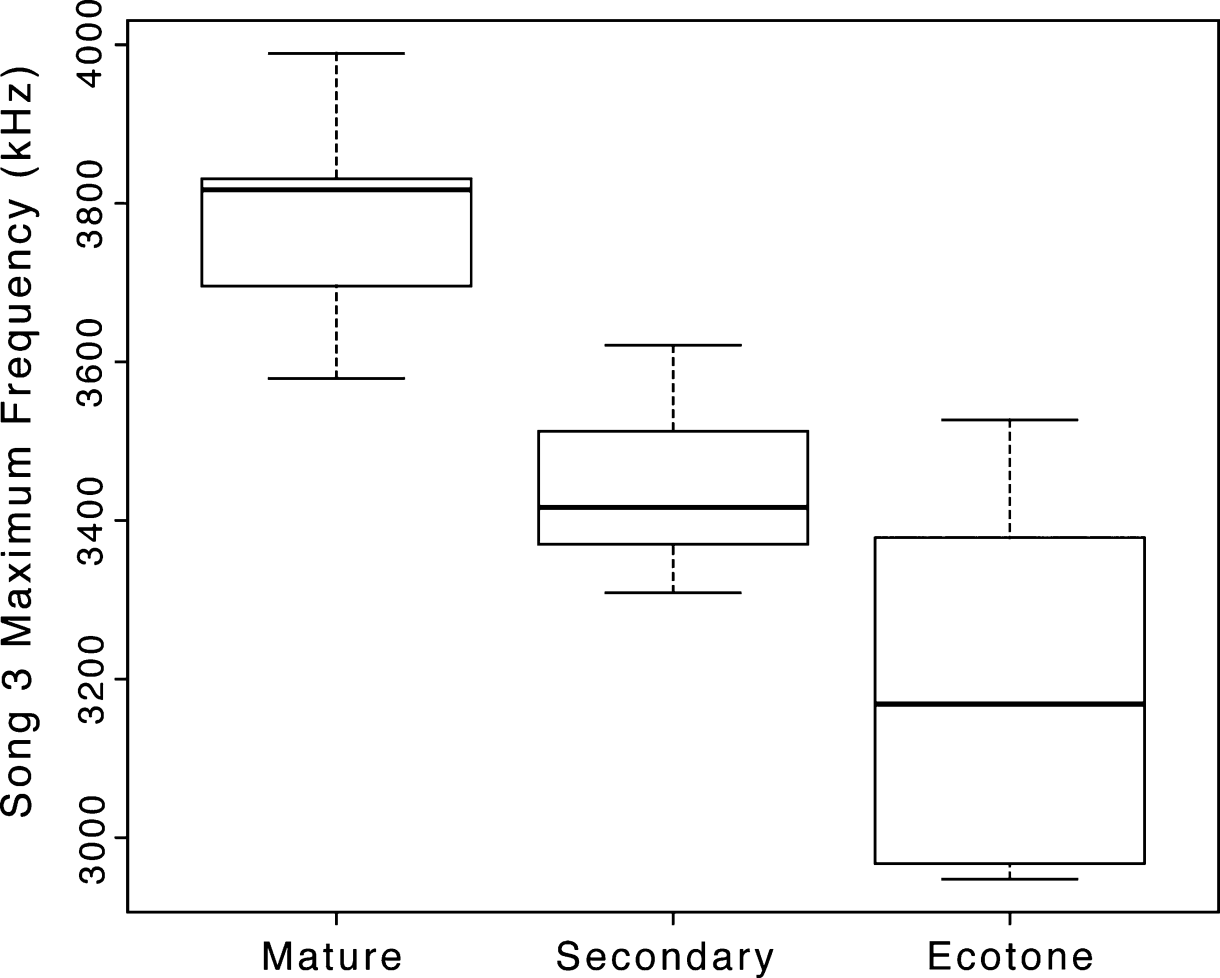
Relationship between forest type and songtype III maximum frequency. For songtype III, little greenbuls sing at a higher maximum in mature forest than secondary (human-altered) forest and ecotone. Dark horizontal lines show median for each forest type, and box boundaries show 25th and 75th percentiles. Whiskers show an estimated two standard deviations from the median. All pairwise comparisons (two-tailed paired *t* tests): mature vs. secondary (*P* = 0.0006), secondary versus ecotone (*P* = 0.003), and mature versus ecotone (*P* = 0.000006) showed significant differences in songtype III maximum frequency. Forest type categories were defined based on Moderate Resolution Imaging Spectroradiometer tree cover data (Smith et al. 2008, 2011).

In addition to habitat correlates with song across undisturbed rainforest and ecotone habitats, we also found songtype III maximum frequency to vary between mature rainforest and anthropogenically altered secondary forest, with birds exhibiting lower frequency song in secondary forest (Fig. 5, Figures S3 and S7). Mature rainforest sites (Fig. 1C) were located between 10 and 30 km from the nearest human settlement, and based on ground, surveys showed little or no signs of human disturbance. Field surveys revealed secondary forest to consist mainly of cacao (*Theobroma cacao*) and coffee (*Coffea* spp.) plantations adjacent to human settlements, with significant disturbance associated with burning, firewood harvesting, and various forms of subsistence agriculture (Smith et al. 2008) (Fig. 1B). These secondary forest frequencies were intermediate between those from mature forests and ecotone habitats, and were consistent with secondary forest birds having vocalizations that were more ecotone like (Fig. 5).

## Discussion

Spatial variables derived from remote-sensing data alone explained up to 66% of the variation in maximum song frequency in songtype III and 45% in songtype IV minimum frequency in the little greenbul. In the case of maximum song frequency in songtype III, we were able to use this high percentage of variation explained to make predictions of the song characteristics of little greenbul songs across a large unsampled region, including Southern Cameroon and parts of the neighboring countries of Equatorial Guinea, Gabon, Republic of Congo, and the Central African Republic. The ability to model and predict a complex trait such as vocal behavior, from remotely sensed data related to key structural differences in habitat, suggests the utility of satellite-based layers for identifying variation in other complex behavioral and phenotypic characteristics of species.

Song characteristics were found to be differentially associated with rainforest, ecotone and secondary forest habitats. Previously, it has been shown that populations of little greenbuls occupying different habitats diverged in acoustic measures of song, with songs of populations in the rainforest having significantly different spectral and temporal characteristics compared to populations in ecotone habitats (Slabbekoorn and Smith 2002b). Transmission properties did not seem to differ in little greenbul-specific forest layers, but there were large differences in the spectral noise profile of the different habitats. Many more noise bands (continuous background noise with characteristic frequencies) within the range relevant to little greenbuls occurred in the rainforest than in the ecotone (Slabbekoorn 2004). Furthermore, more prominent noise bands occur high in their song frequency range, which may yield a general selection pressure for little greenbuls in the rainforest to sing at lower minimum frequencies (Slabbekoorn and Smith 2002b). However, noise bands vary in relative loudness and bandwidth and sometimes narrow slits in the noise profile can lead to spectrally local selection pressures that are upward or downward (Slabbekoorn 2004). Congruently, we suggested that some song variation, such as observed for the maximum frequency of songtype III, may be related to prominent noise bands in the rainforest driving the frequency use upward. In the current study, we confirmed broad-scale habitat differences in songtype III maximum frequency (Fig. 5) and were also able to document significant differences in song characteristics even within forested sites.

Within forested sites themselves, the greatest observed differences in song characteristics occur between mature and secondary forest types. Song characteristics from secondary forests were intermediate between the values of rainforest and ecotone forest and may reflect noise produced with intermediate frequency values by the surrounding animal community (Lawton et al. 1988; Slabbekoorn 2004). Perhaps due to the lack of insect species generating sounds that compete within the same frequency bands as greenbuls, parts of the noise spectrum may become acoustically more similar to the open forest/savannah habitat found in the ecotone regions of northern Cameroon (Slabbekoorn 2004; Smith et al. 2008). In a previous analysis, we did not find significant spectral variation, but temporal variation in little greenbul song did also reveal intermediate measures for human disturbed secondary forest, which were intermediate between those of mature rainforest and ecotone forest (Smith et al. 2008). Although similar sound-transmission properties between ecotone and forest have been previously reported (Slabbekoorn and Smith 2002b), future work should examine the role of forest structure in altering acoustic signals in greater depth. The concept that signals degradation varies according to forest type or between forest and ecotone habitats remains a possibility.

The differences in song characteristics between disturbed and undisturbed forests suggest that human-altered habitats have induced behavioral changes in this species that have been rapid, consistent with findings from studies of some urban bird species (Slabbekoorn and den Boer-Visser 2006; Mockford and Marshall 2009). Exactly, how rapidly these changes have occurred is unclear, but we estimate the conversion of mature to secondary forest in the region was well underway 100 years ago (Bates 1930; Merfield 1957). Whether little greenbuls differentially perceive songs from mature and human-altered habitats, as they do between ecotone and forest habitats (Kirschel et al. 2011), remains to be examined. Despite evidence of significant gene flow between habitats (Smith et al. 1997), evidence suggests genetic divergence between mature and secondary forest populations (Smith et al. 2008). Recent research has shown that human-altered environments can lead to changes in avian song (Slabbekoorn and Peet 2003; Slabbekoorn and den Boer-Visser 2006; Halfwerk and Slabbekoorn 2009; Kirschel et al. 2009a), and once these songs have been modified, they may provide the initial steps to premating reproductive isolation. Rather than songs in secondary forest arising *in situ*, it is also possible that individuals from the ecotone might be colonizing secondary forest. Future work seeks to distinguish between these two possibilities.

We have shown it is possible to accurately predict geographic variation in a behavioral trait, in this case, bird song, using remote-sensing data. These types of spatially continuous predictions for a complex behavior such as song allow for predictions to be made in either inaccessible or infeasible regions, and greatly add in determining how behavioral variation is distributed across a landscape. This approach also affords the opportunity to identify geographic areas of variance in behavioral traits, corresponding to either physical barriers to dispersal, or in the case of little greenbuls, less obvious anthropogenic habitat changes that are associated with distinct behavioral differences within species. While our approach was able to use ground-truthed locations sampled during a subsequent trip to the region, it should be noted that models attempting to relate ecological variation to complex traits can be expected to perform only as well as the original data used as input. Particular importance should be placed on assuring that sampled locations cover as broad an ecological context as possible; niche models often lose their ability to predict in regions that are ecologically speaking represent extremes as compared to locations used as input in training (Figure S9). Additionally, some physical characteristics of song seemed to show little to no correlation with variables capturing environmental heterogeneity (for instance, songtype IV peak frequency), suggesting that either local adaptation and/or phylogenetic constraints should be considered in an attempt to fully understand the factors contributing to behavior variation among populations.

Because behavioral traits are typically studied at relatively small spatial scales, we believe our approach, and extensions of it, has the potential of opening a new frontier for the use of remote-sensing data to investigate animal behavior in both natural and human-altered habitats at very large spatial scales. The observed and predicted variation in song documented in this study could ultimately be driven by a number of environmental factors, including forest structure (Wiley and Richards 1982), acoustic competition with other species (Kirschel et al. 2009a,b) or background noise (Slabbekoorn and Smith 2002a; Price 2008). Previous studies on urban noise have shown that birdsong frequencies may shift at various time scales and become correlated with local noise levels and profiles (Patricelli and Blickley #eva12072-bib-1000^2006^; Slabbekoorn 2013). It is also possible that birds are adjusting song characteristics to varying ecological conditions (Medina and Francis 2012), through behavioral plasticity. Future work will be required to determine the extent to which song differences are the result of plasticity or adaptive divergence.

In conclusion, our approach provides a link between large-scale remotely sensed environmental variables and on-the-ground measurements of behavior that can have both evolutionarily important impacts and conservation implications. Further investigations and experiments are required to test the mechanistic hypotheses about correlations between greenbul song frequencies and habitat-dependent ambient noise profiles. However, the significant acoustic variation across habitats, including those between human-degraded secondary and mature rainforest, has the potential to lead to localized adaptation that may affect reproductive divergence and gene flow. Our approach also has the potential to help identify areas of dynamic biotic change, important for investigating a wide range of biotic processes. Identifying biodiversity hotspots for conservation prioritization and contact zones between evolutionary distinct populations can be difficult and costly. Therefore, we hope to stimulate future studies that will yield fundamental insights into evolutionary processes as well as valuable contributions to conservation.

## Data sharing/Archiving

Data deposited in the Dryad repository: doi:10.5061/dryad.c1q5h

## Supporting Information

**Figure S1.** Tree regressions used to describe variation in song characteristics in little greenbuls (songtype I).

**Figure S2.** Tree regressions used to describe variation in song characteristics in little greenbuls (songtype II).

**Figure S3.** Tree regressions used to describe variation in song characteristics in little greenbuls (songtype III).

**Figure S4.** Tree regressions used to describe variation in song characteristics in little greenbuls (songtype IV).

**Figure S5.** Maximum variation explained and importance scores for each environmental variable used as input to describe songtype I characteristics in little greenbuls in Cameroon under random forest.

**Figure S6.** Maximum variation explained and importance scores for each environmental variable used as input to describe songtype II characteristics in little greenbuls in Cameroon under random forest.

**Figure S7.** Maximum variation explained and importance scores for each environmental variable used as input to describe songtype III characteristics in little greenbuls in Cameroon under random forest.

**Figure S8.** Maximum variation explained and importance scores for each environmental variable used as input to describe songtype IV characteristics in little greenbuls in Cameroon under random forest.

**Table S1.** Site names, habitat type and coordinates where little greenbul songs were recorded in Cameroon.

**Table S2.** Sampling sites, number of individuals from which recordings of songs were analyzed, and sampling dates.
