# Supplementary material for: Predicting bird song from space
Source: Evol Appl. 2013 May 8;6(6):865–74. doi: 10.1111/eva.12072 (PMC3779089; doi:10.1111/eva.12072)
Supplement: Supplementary file 1 [file eva0006-0865-SD1.doc]

**Supporting Information**

**Predicting Bird Song from Space**

Thomas B. Smith, Ryan J. Harrigan, Alexander N. G. Kirschel, Wolfgang Buermann, Sassan Saatchi, Daniel T. Blumstein, Selvino R. de Kort, Hans Slabbekoorn

**List of Contents**

Supplementary Figs 1 through 8

Supplementary Tables 1 and 2


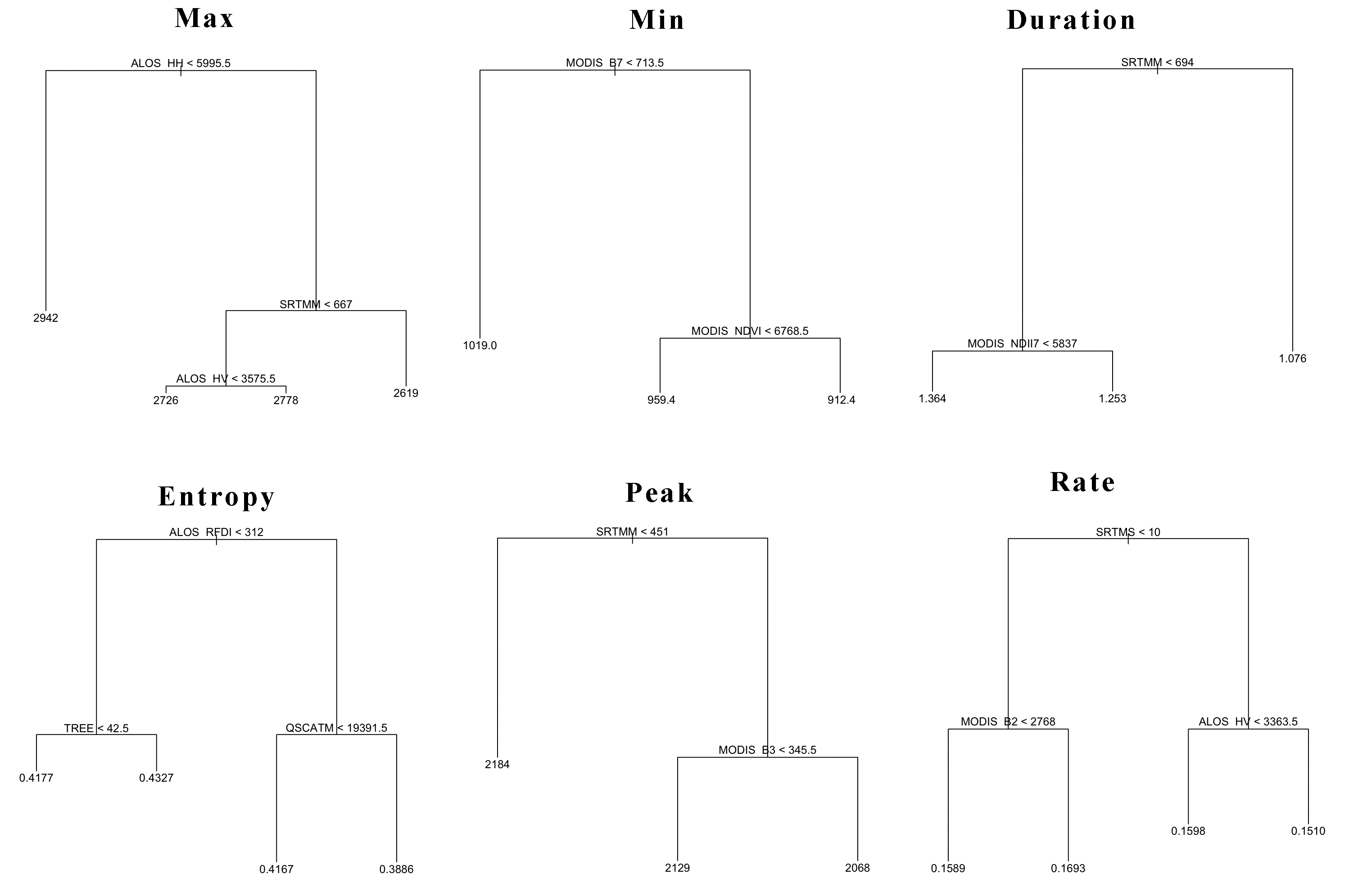


Figure S1. Tree regressions used to describe variation in song characteristics in little greenbuls (songtype I). Variables used to split frequency values are indicated at each node (with higher values of each variable represented towards the right-side branch). Branch lengths correspond to the amount of deviance in prevalence explained by the variable at that node. Numbers at terminal nodes represent average song characteristic value within that group.

**
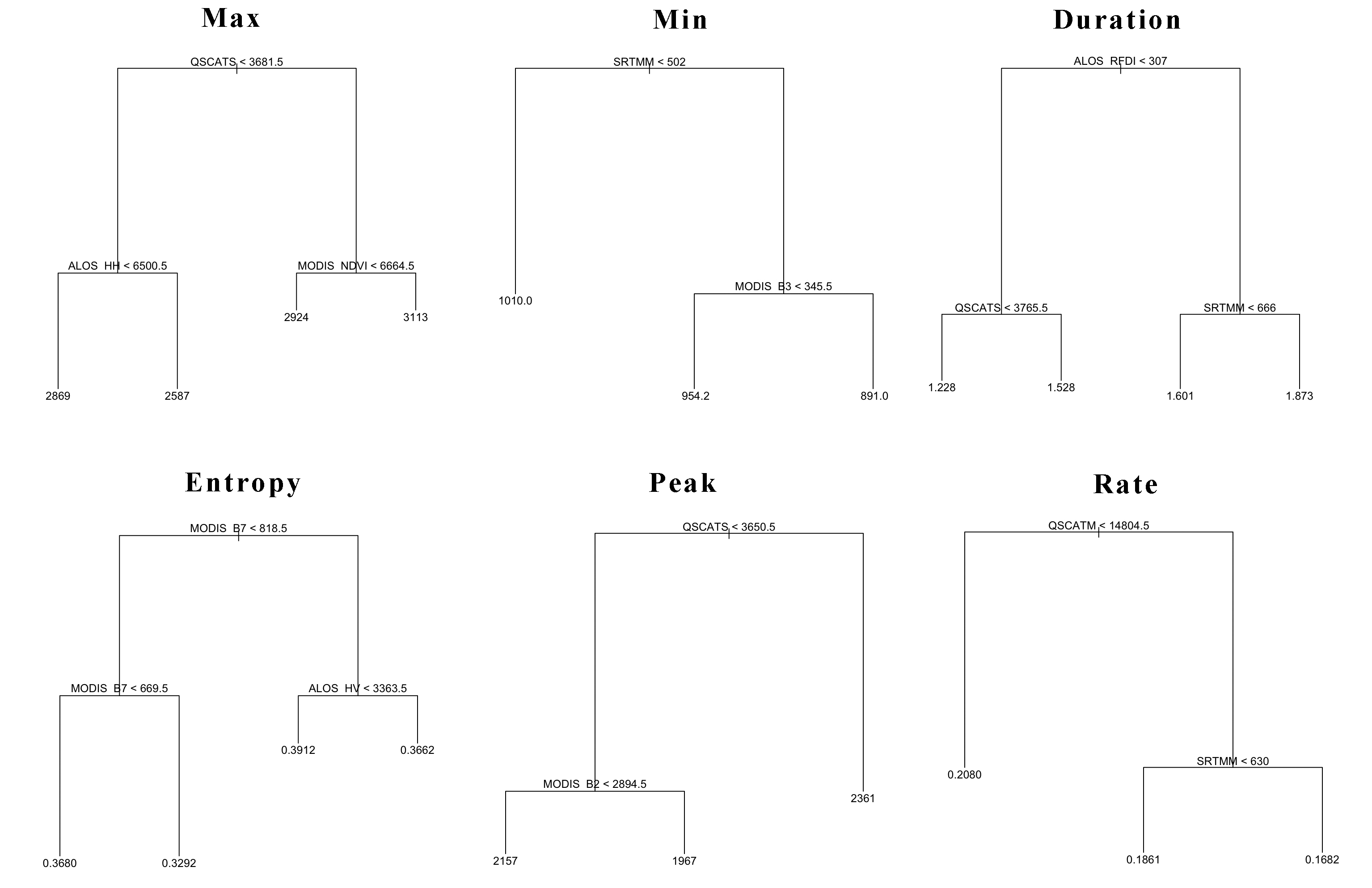
**

Figure S2. Tree regressions used to describe variation in song characteristics in little greenbuls (songtype II). Variables used to split frequency values are indicated at each node (with higher values of each variable represented towards the right-side branch). Branch lengths correspond to the amount of deviance in prevalence explained by the variable at that node. Numbers at terminal nodes represent average song characteristic value within that group.


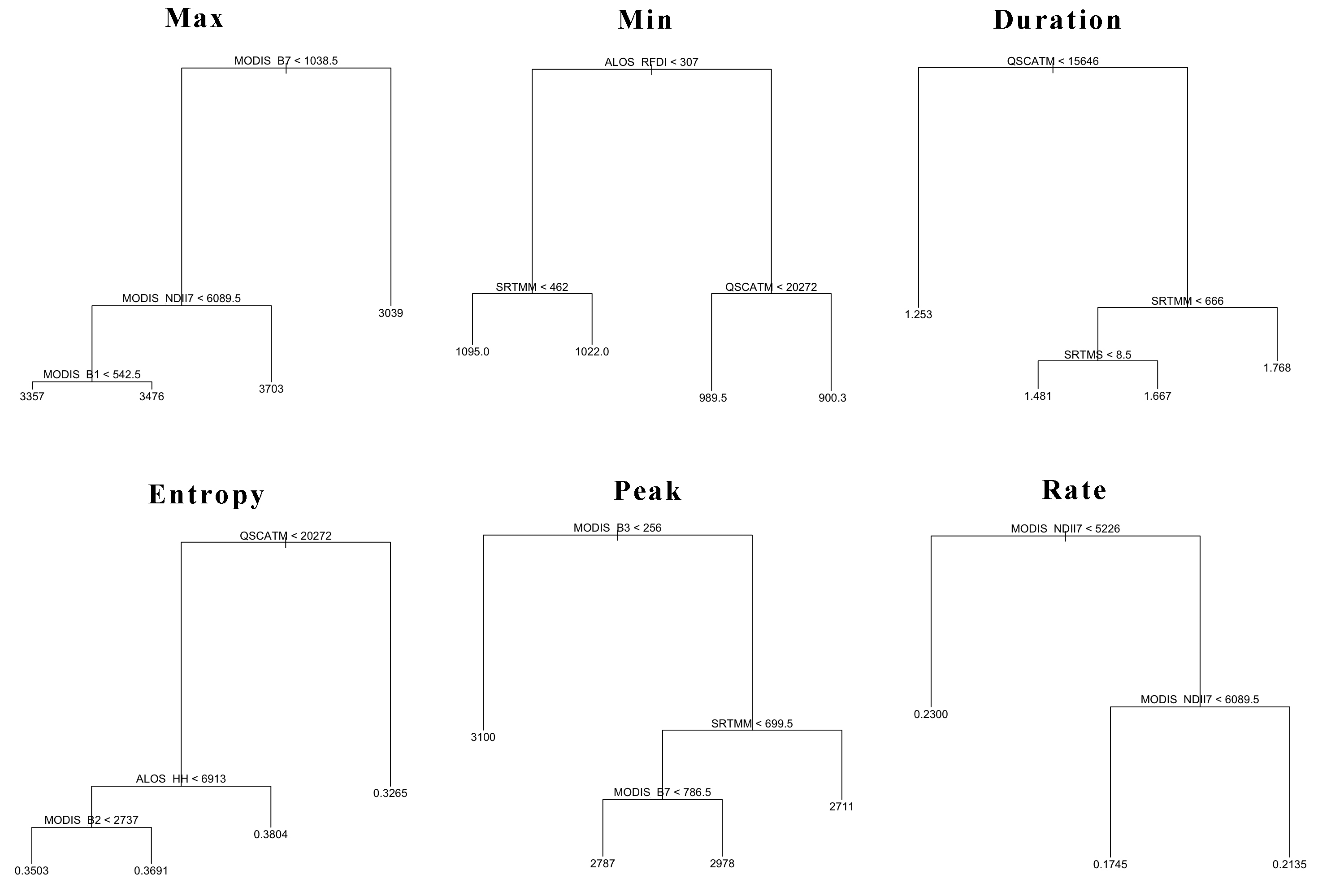


Figure S3. Tree regressions used to describe variation in song characteristics in little greenbuls (songtype III). Variables used to split frequency values are indicated at each node (with higher values of each variable represented towards the right-side branch). Branch lengths correspond to the amount of deviance in prevalence explained by the variable at that node. Numbers at terminal nodes represent average song characteristic value within that group.


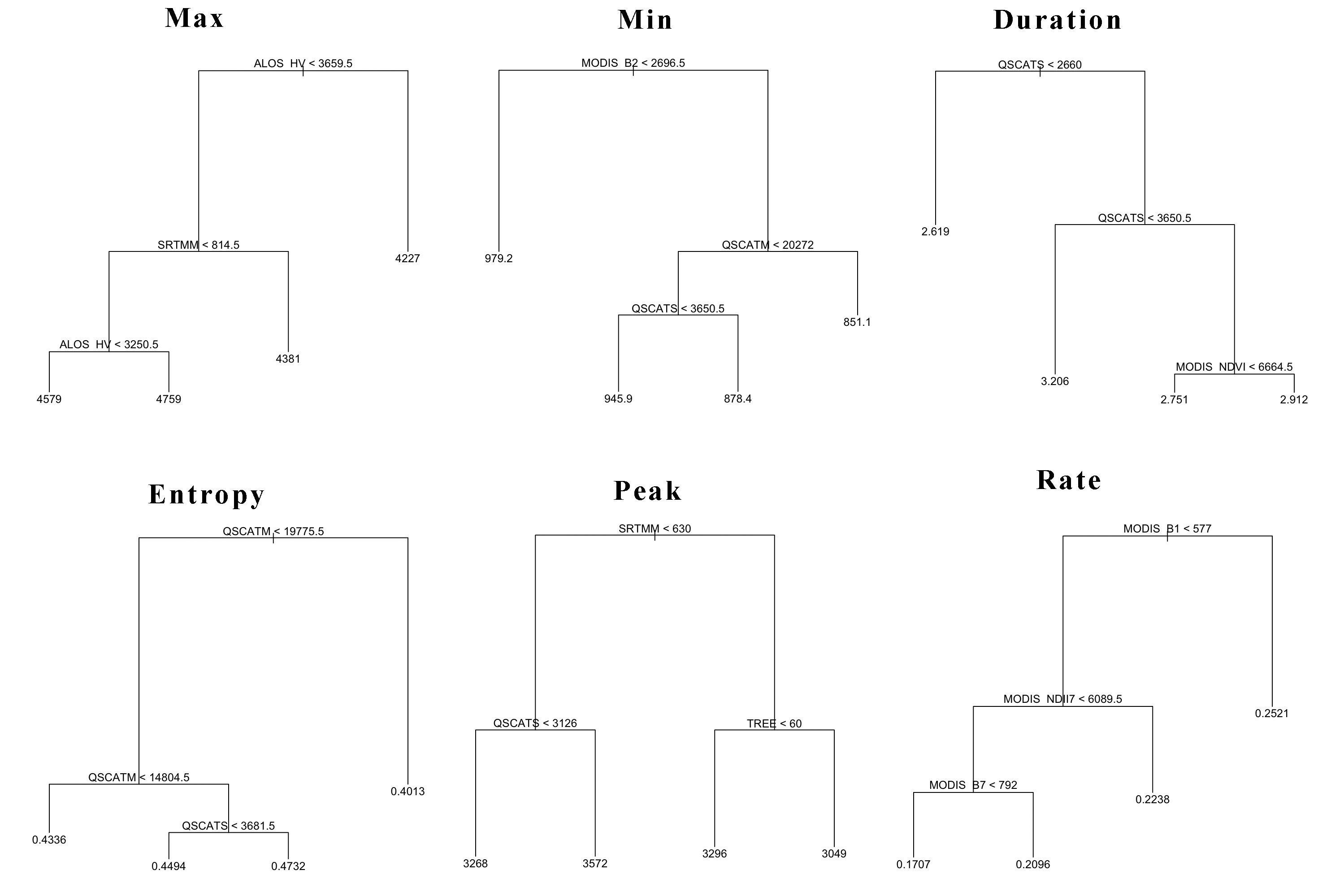


Figure S4. Tree regressions used to describe variation in song characteristics in little greenbuls (songtype IV). Variables used to split frequency values are indicated at each node (with higher values of each variable represented towards the right-side branch). Branch lengths correspond to the amount of deviance in prevalence explained by the variable at that node. Numbers at terminal nodes represent average song characteristic value within that group.

**
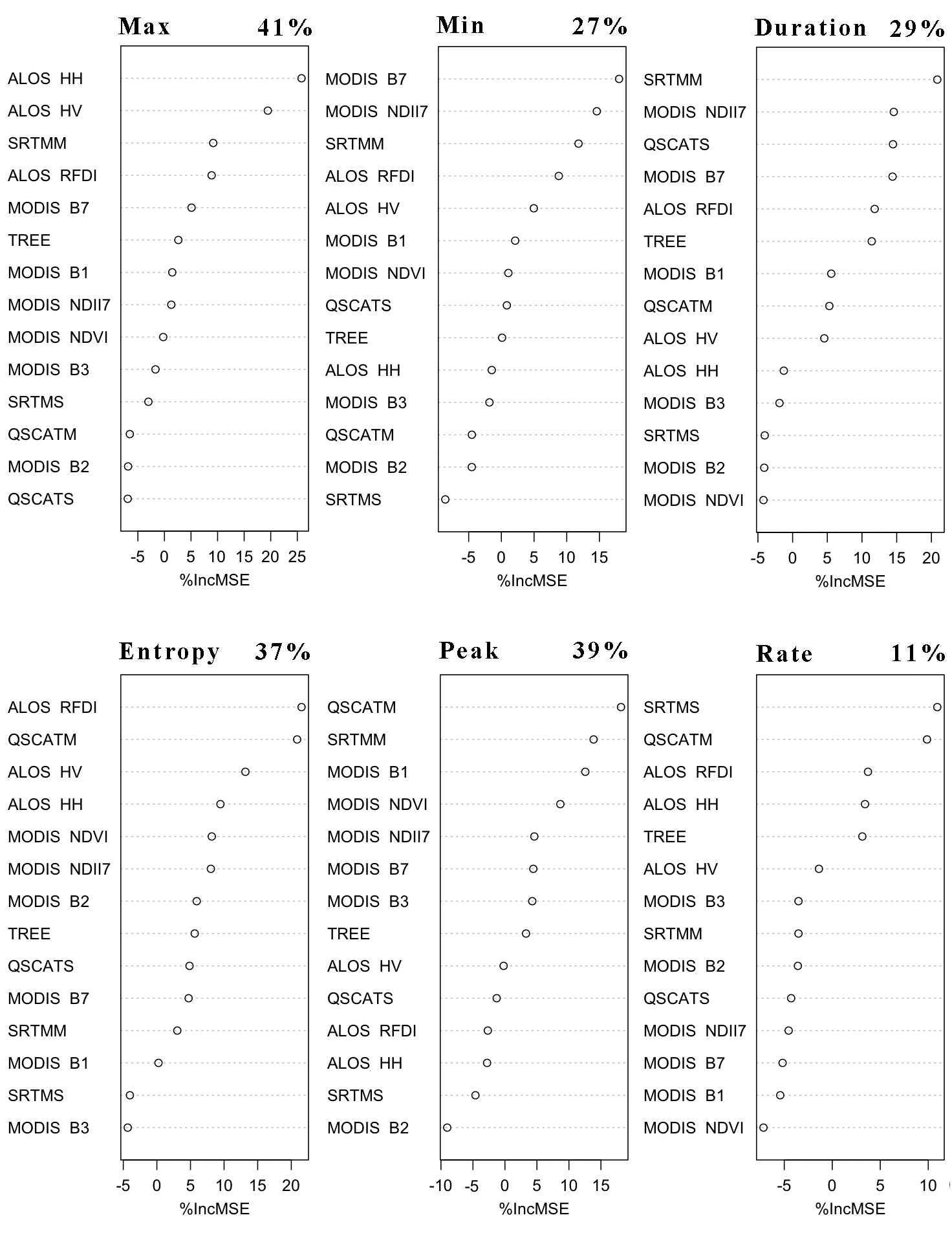
**

Figure S5. Maximum variation explained and importance scores for each environmental variable used as input to describe songtype I characteristics in little greenbuls in Cameroon under random forest. Negative values indicate that with the removal of that particular variable, the percent increase in mean square error decreases, and identifies that variable as a poor predictor in the model. See Supplementary Table 2 for description of predictor variables.

**
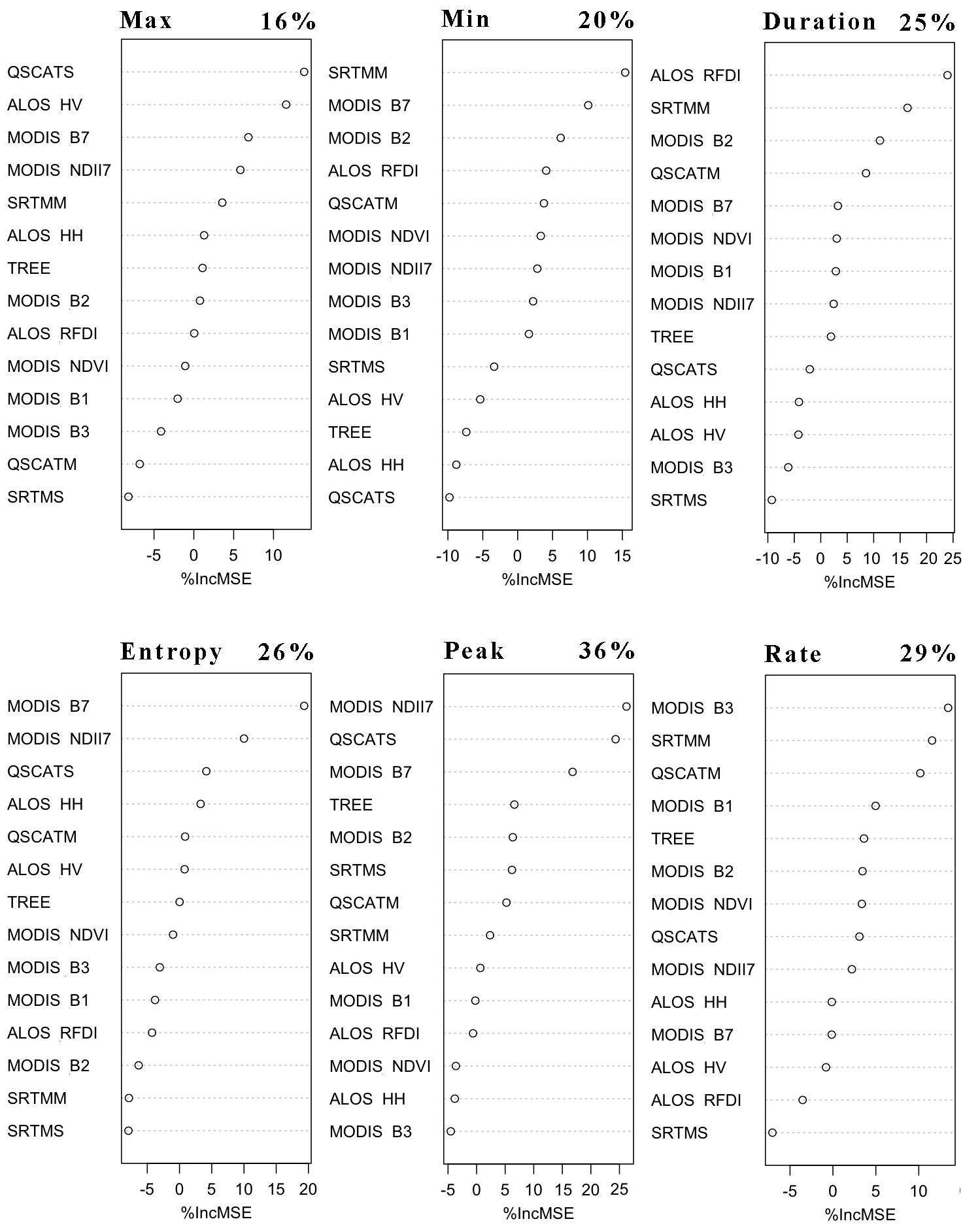
**

Figure S6. Maximum variation explained and importance scores for each environmental variable used as input to describe songtype II characteristics in little greenbuls in Cameroon under random forest. Negative values indicate that with the removal of that particular variable, the percent increase in mean square error decreases, and identifies that variable as a poor predictor in the model.

**
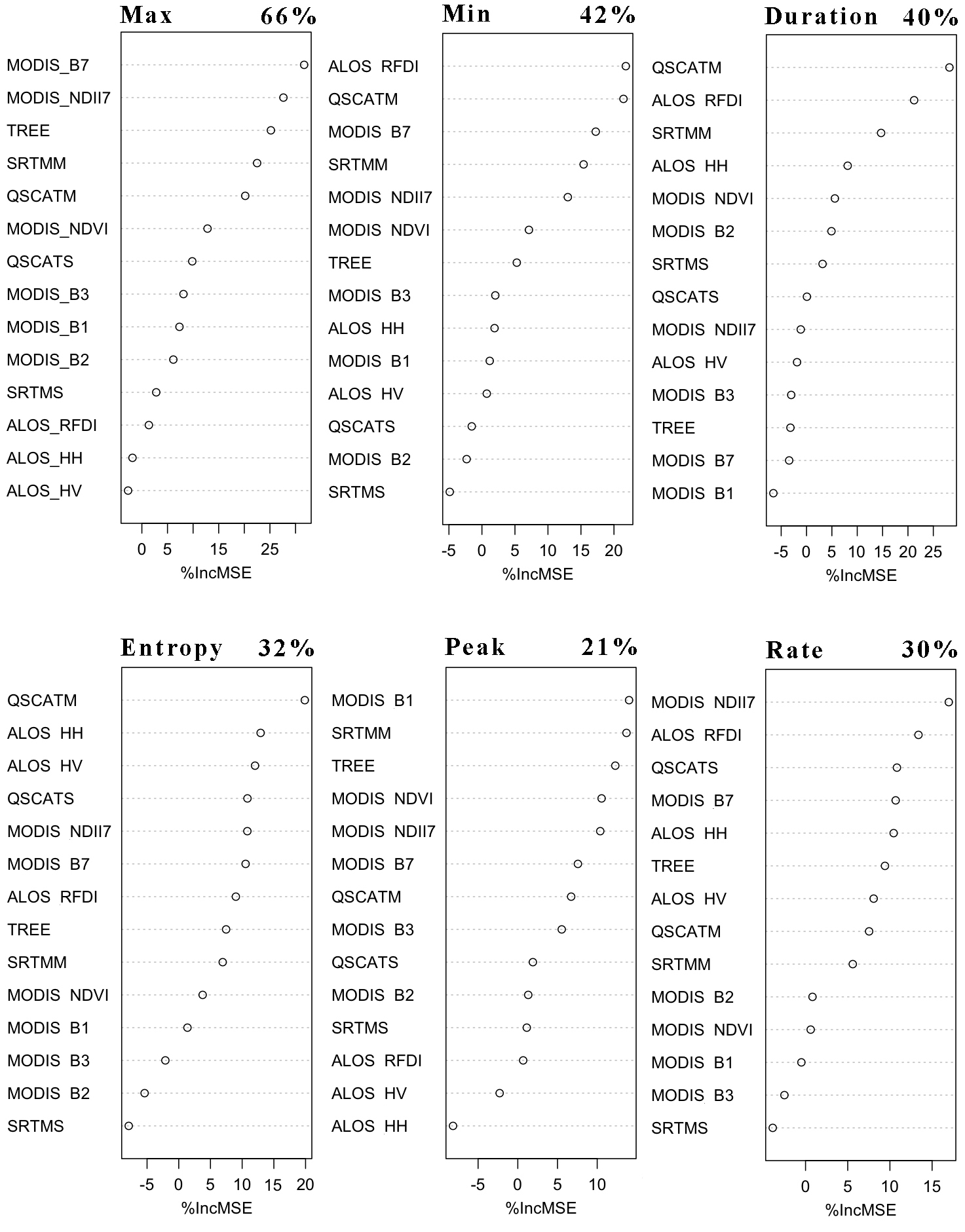
**

Figure S7. Maximum variation explained and importance scores for each environmental variable used as input to describe songtype III characteristics in little greenbuls in Cameroon under random forest. Negative values indicate that with the removal of that particular variable, the percent increase in mean square error decreases, and identifies that variable as a poor predictor in the model.


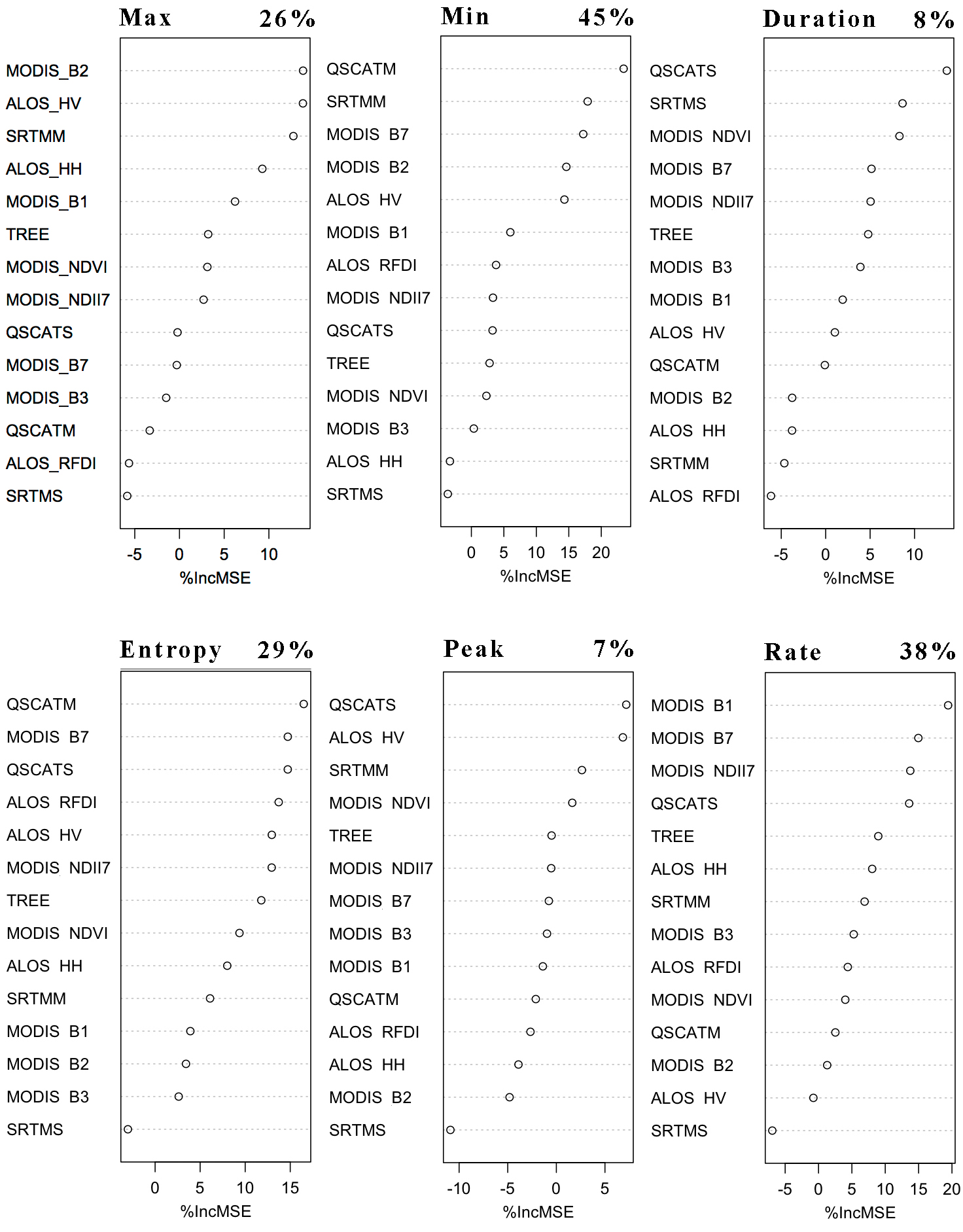


Figure S8. Maximum variation explained and importance scores for each environmental variable used as input to describe songtype IV characteristics in little greenbuls in Cameroon under random forest. Negative values indicate that with the removal of that particular variable, the percent increase in mean square error decreases, and identifies that variable as a poor predictor in the model.

Table S1. Site names, habitat type and coordinates where little greenbul songs were recorded in Cameroon.

Site Forest Type Latitude Longitude

­­­­­­­–––––––––––––––––––––––––––­­­­­­­­­­–––––––––––––––––––––––––––––––––––––––––––––––––––––––––––––––––––––

Bertoua Mature 4.716650 N 13.616700 E

Betare Oya Ecotone 5.563392 14.092340

Bitye Secondary 3.008113 12.357579

Campo Secondary 2.349695 9.828976

Dja Mature 3.190339 12.813070

Etome Secondary 4.043516 9.118556

Kompe Secondary 3.538070 12.835310

Kousse Ecotone 4.449388 11.545028

Kribi Secondary 2.727870 9.871403

Lobeke Mature 2.301434 15.759030

Mbassako Mature 3.173370 12.818260

Meiganga Ecotone 6.515415 14.316123

Ndibi Secondary 3.777615 12.205326

Ndikinimeki Ecotone 4.762950 10.839345

Ndogmem Secondary 4.513177 10.365677

Ngoundaba Ecotone 7.121331 13.701215

Nguti Mature 5.333015 9.466714

Nkoleon Secondary 2.395212 10.044972

Nkwouak Secondary 3.856609 13.324783

Obala Ecotone 4.184919 11.540066

Wakwa Ecotone 7.224110 13.545315

Yoko Ecotone 5.540898 12.322025

Zaokom Secondary 3.138006 11.825644

Zoebefam Mature 2.659740 13.379642

–––––––––––––––––––––––––––­­­­­­­­­­–––––––––––––––––––––––––––––––––––––––––––––––––––––––––––––––––––––

Table S2. Sampling sites, number of individuals from which recordings of songs were analyzed, and sampling dates.

Site No. individuals analyzed Sampling dates

­­­­­­­–––––––––––––––––––––––––––­­­­­­­­­­–––––––––––––––––––––––––––––––––––––––––––––––––––––––––––––––––––––

Bertoua 6 14 February 1999

Betare Oya 5 19 June 1998, 16 Feb 1999

Bitye 5 20 August 2009

Campo 5 1 August 2007

Dja 5 11 April 1999

Etome 5 22-24 March 2007

Kompe 5 6 August 2009

Kousse 5 26-27 June 1999

Kribi 5 25-29 July 2007; 4-6 June 1999,

Lobeke 5 15-June 1998

Mbassako 5 27 May 1998

Meiganga 5 28 February 1999

Ndibi 5 27 June - 5 July- 2005; 10 June 1999

Ndikinimeki 5 5 December 1998

Ndogmem 4 21-24 July 2007

Ngoundaba 5 11-14 March 2007; 26-28 Feb 1999

Nguti 5 19-31 March 1999

Nkoleon 4 30-31 July 2007

Nkwouak 5 7-12 July 2005; 11-12 June 1998

Obala 5 6-8 March 2007; 7 June 1998

Wakwa 5 16-18 March 2007

Yoko 5 9-12 March 1999

Zaokom 4 21 August 2009

Zoebefam 4 24-28 July 2005; 6-7 May 1999

–––––––––––––––––––––––––––­­­­­­­­­­–––––––––––––––––––––––––––––––––––––––––––––––––––––––––––––––––––––
